# Characteristics and outcomes of COVID-19 in heart transplantation recipients in the Netherlands

**DOI:** 10.1007/s12471-022-01720-9

**Published:** 2022-09-08

**Authors:** S. A. Muller, O. C. Manintveld, M. K. Szymanski, K. Damman, M. G. van der Meer, K. Caliskan, L. W. van Laake, M. I. F. J. Oerlemans

**Affiliations:** 1 Department of Cardiology, Division Heart and Lung, University Medical Centre Utrecht, Utrecht University, Utrecht, The Netherlands; 2 Department of Cardiology, Thorax Centre, Erasmus Medical Centre, Rotterdam, The Netherlands; 3 Erasmus Medical Centre Transplant Institute, Rotterdam, The Netherlands; 4 Department of Cardiology, University Medical Centre Groningen, University of Groningen, Groningen, The Netherlands

**Keywords:** Heart transplantation, HTx, COVID-19, Coronavirus disease 2019

## Abstract

**Background:**

Immunocompromised patients are at high risk of complicated severe acute respiratory coronavirus 2 infection. The aim of this retrospective study was to describe the characteristics and outcomes of heart transplantation (HTx) recipients with coronavirus disease 2019 (COVID-19) in the Netherlands.

**Methods:**

HTx patients from one of the three HTx centres in the Netherlands with COVID-19 (proven by positive reverse-transcription polymerase chain reaction or serology test result) between February 2020 and June 2021 were included. The primary endpoint was all-cause mortality and the secondary endpoint was disease severity.

**Results:**

COVID-19 was diagnosed in 54/665 HTx patients (8%), with a mean (± standard deviation (SD)) time after HTx of 11 ± 8 years. Mean (± SD) age was 53 ± 14 years and 39% were female. Immunosuppressive therapy dosage was reduced in 37% patients (20/54). Hospitalisation was required in 39% patients (21/54), and 13% patients (7/54) had severe COVID-19 (leading to intensive care unit (ICU) admission or death). In-hospital mortality was 14% (3/21), and all-cause mortality was 6%. Compared with patients with moderate COVID-19 (hospitalised without ICU indication), severe COVID-19 patients tended to be transplanted earlier and had a significantly higher mean (± SD) body mass index (26 ± 3 vs 30 ± 3 kg/m^2^, *p* = 0.01). Myocardial infarction, cellular rejection and pulmonary embolism were observed once in three different HTx patients.

**Conclusion:**

HTx patients were at increased risk of complicated COVID-19 with frequent hospitalisation, but the all-cause mortality was substantially lower than previously described (7–33%).

**Supplementary Information:**

The online version of this article (10.1007/s12471-022-01720-9) contains supplementary material, which is available to authorized users.

## What’s new?

- Heart transplantation (HTx) recipients were at increased risk of complicated COVID-19 with frequent hospitalisation.
- HTx patients with severe COVID-19 were older, more often had underlying ischaemic heart failure and more often presented with fever.
- In our cohort, the all-cause mortality rate (6%) was substantially lower than previously reported (7–33%), which could be explained by the availability of routine COVID-19 testing in the Netherlands and contact tracing provided by the Dutch Municipal Health Service.

## Introduction

Coronavirus disease 2019 (COVID-19) has caused a pandemic for over 2 years, affecting nearly 2 million people in the Netherlands, with an overall mortality rate of 0.3% as of 10 May 2022 [1]. Multiple patient populations have been identified as having a high risk of developing complicated severe acute respiratory coronavirus 2 (SARS-CoV-2) infection, resulting in a higher rate of hospitalisation, increased need for mechanical ventilation support and higher risk of all-cause mortality.

Although it has been hypothesised that immunocompromised patients are protected from cytokine storm, both immunocompromised patients and patients with pre-existing cardiovascular diseases are considered to be at high risk of complicated COVID-19 [2, 3]. Heart transplantation (HTx) recipients are part of both groups, having multiple cardiovascular risk factors, such as hypertension and cardiac allograft vasculopathy (CAV), as well as being immunocompromised to avoid rejection. Moreover, increasing evidence suggests that the humoral and cellular responses after COVID-19 vaccination in patients after solid organ transplantation are significantly impaired, leading to a reduced antibody response [4–6]. Therefore, even in the COVID-19 vaccination era, HTx patients are considered a high-risk population [4–6].

Previous studies have shown COVID-19-related mortality rates of up to 7–33% in HTx patients [2, 7–15]. However, these studies mainly consisted of HTx patients requiring hospitalisation as patients were included after consultation in the emergency department. Patients with COVID-19 not requiring hospitalisation might therefore have been missed, which could have led to overestimation of the mortality rate. In the Netherlands, routine testing and contact tracing are available, allowing for identification of COVID-19 patients who are not necessarily in need of hospitalisation.

In this study, we aimed to describe the characteristics and outcomes of COVID-19 in HTx patients in a situation where routine testing and contact tracing were available.

## Methods

### Study population

In this multicentre, retrospective, observational study, we included HTx patients from all three HTx centres in the Netherlands with proven COVID-19 between 27 February 2020 and 1 June 2021. Proven COVID-19 was defined as a positive reverse-transcription polymerase chain reaction (RT-PCR) test result or a positive serology test result.

### Data collection and stratification

Data were extracted from electronic health records and included information on cardiovascular risk factors, medication, aetiology of heart failure, time after HTx, CAV and history of cellular rejection one year or longer prior to COVID-19. Cardiovascular risk factors were defined according to the corresponding guidelines [16–18]. Furthermore, data on symptoms during COVID-19, laboratory results at presentation, immunosuppressive regiment during COVID-19, administration of dexamethasone, nadroparin and/or antiviral medication, and duration of hospitalisation were collected.

The study was approved by the local medical ethics committees of the three transplant centres. Furthermore, all HTx recipients provided written informed consent for collection of clinical data as part of a national ongoing quality improvement programme.

### Endpoints

The primary endpoint of this study was all-cause mortality, and the secondary endpoint was severity of COVID-19.

### Statistical analysis

For descriptive statistics, data were stratified by severity of COVID-19, defined as mild (not hospitalised), moderate (hospitalised, no indication for intensive care unit (ICU) admission) and severe (hospitalised with ICU admission or death). Nominal variables were expressed as number and percentage, whereas continuous variables were expressed as mean ± standard deviation (SD) or median and interquartile range (IQR) depending on distribution.

Statistical analyses of binary variables were performed using Pearson’s chi-square test to compare three groups and chi-square test, or Fisher’s exact test as appropriate, to compare two groups. For continuous variables, one-way ANOVA or, if not normally distributed, Kruskal-Wallis test was carried out to compare three groups, and an independent *t*-test or a Mann-Whitney U test, depending on distribution, was use to compare two groups.

Data were analysed using SPSS Statistics for Mac, version 27 (IBM Corp., Armonk, NY, USA). A *p*-value < 0.05 was considered statistically significant.

## Results

### Patient characteristics

**Fig. 1:**
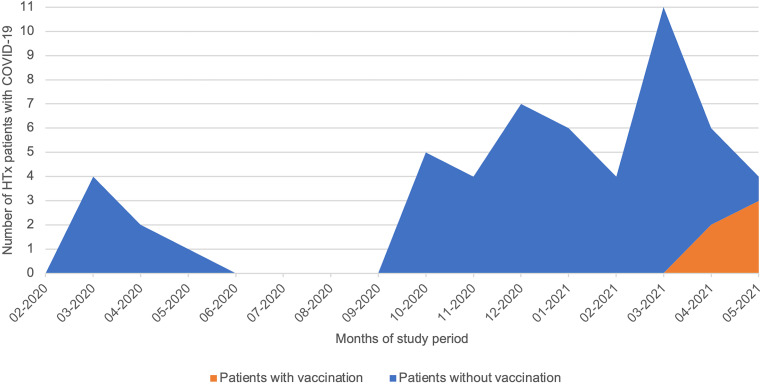
Number of heart transplantation (*HTx*) patients with COVID-19 by month and vaccination status

Of the 665 HTx patients included between 27 February 2020 and 1 June 2021, 54 (8%) had proven COVID-19. Fig. 1 shows the number of COVID-19 patients by month and vaccination status. Characteristics of the 54 patients during COVID-19 are shown in Tab. 1. Mean (± SD) age was 53 ± 14 years, mean (± SD) time after HTx was 11 ± 8 years and 39% were female. RT-PCR and serology test results were positive for SARS-CoV‑2 in 52 and 2 patients, respectively. The two patients with positive serology had symptoms indicative of prior COVID-19 and were not vaccinated at the time of the test. Symptoms at presentation were upper airway complaints (63%), fatigue (61%), declined exercise tolerance (52%), fever (48%), muscle ache (39%), gastro-intestinal symptoms (22%) and anosmia (15%) (see Table S1 in Electronic Supplementary Material). Mild, moderate and severe COVID-19 occurred in 33 (61%), 14 (26%) and 7 patients (13%), respectively (Tab. 1). Of all included HTx patients, 5 patients (9%) were (partially) vaccinated against COVID-19 at the time of diagnosis, of whom 4 required hospitalisation.

**Table 1:** Patient characteristics during COVID-19, overall and stratified by severity of COVID-19

	Total	Severity of COVID-19		
Variable	Patients (*n* = 54)	Mild (*n* = 33)	Moderate (*n* = 14)	Severe (*n* = 7)
Age, years^a^	53 ± 14	48 ± 13	58 ± 13	63 ± 13
Time after HTx, years	11 ± 8	10 ± 8	9.1 ± 5	16 ± 9
Female	21 (39)	15 (45)	4 (29)	2 (29)
BMI, kg/m^2b^	26 ± 5	26 ± 5	26 ± 3	30 ± 3
*Aetiology heart failure*				
– DCM	20 (37)	13 (39)	6 (43)	1 (14)
– HCM	7 (13)	6 (18)	1 (7)	0
– Ischaemic^a,b^	12 (22)	5 (15)	2 (14)	5 (71)
– Congenital	4 (7)	1 (3)	3 (21)	0
– Myocarditis	2 (4)	2 (6)	0	0
– Genetic	2 (4)	2 (6)	0	0
– Sarcoidosis	2 (4)	1 (3)	1 (7)	0
– Toxic	2 (4)	2 (6)	0	0
– ARVC	3 (6)	1 (3)	1 (7)	1 (14)
*Cardiovascular risk factors*				
– Diabetes mellitus	14 (26)	6 (18)	5 (36)	3 (43)
– Hypertension	45 (83)	27 (81)	11 (79)	7 (100)
– Hypercholesterolaemia	48 (89)	28 (85)	13 (93)	7 (100)
– COPD	3 (6)	1 (3)	1 (7)	1 (14)
– Dialysis	4 (7)	2 (6)	2 (14)	0
– CAV	20 (37)	12 (36)	3 (21)	5 (71)
– Rejection in the past	20 (37)	9 (27)	6 (43)	5 (71)
*Immunosuppressive medication*				
– Mycophenolic acid	32 (59)	19 (58)	9 (64)	4 (57)
– Tacrolimus	46 (85)	30 (91)	12 (86)	4 (57)
– Ciclosporin	8 (15)	3 (9)	2 (14)	3 (43)
– Azathioprine	2 (4)	0	1 (7)	1 (14)
– Prednisone	25 (46)	14 (42)	6 (43)	5 (71)
– Everolimus	9 (17)	7 (21)	2 (14)	0
– Sirolimus	1 (2)	1 (3)	0	0
Dosage of immunosuppressive medication lowered^c^	22 (41)	6 (18)	10 (71)	6 (86)
*COVID-specific medication*				
– Dexamethasone	8/21 (38)	–	5/14 (36)	3/7 (43)
– Nadroparin	8/21 (38)	–	5/14 (36)	3/7 (43)
– Antiviral medication^b^	7/21 (33)	–	2/14 (14)	5/7 (71)
– Interleukin‑6 antagonist	1/21 (5)	–	0	1/7 (14)
*Hospitalisation characteristics*				
– Time in hospital, days^b^	8.0 (3.5–14.0)	–	6 (3.0–10.0)	15 (8.0–22.0)
– Time in clinical ward, days	6.5 (3.3–10.8)	–	6 (3.0–10.0)	10 (5.5–16.5)
– Time in ICU, days	8.0 (5.5–16.5)	–	–	8.0 (5.5–16.5)

Data are mean ± standard deviation, *n* (%) or median (interquartile range)

*HTx* heart transplantation, *BMI* body mass index, *DCM* dilated cardiomyopathy, *HCM* hypertrophic cardiomyopathy, *ARVC* arrhythmogenic right ventricular cardiomyopathy, *COPD* chronic obstructive pulmonary disease, *CAV* cardiac allograft vasculopathy, *ICU* intensive care unit

^a^0.01 > *p* ≥ 0.001 (comparison between mild, moderate and severe disease groups)

^b^0.05 > *p* ≥ 0.01 (comparison between moderate and severe disease groups)

^c^*p* < 0.001 (comparison between mild, moderate and severe disease groups)

Compared with patients with less severe COVID-19, those with severe disease were older at time of infection (*p* = 0.007), more often had ischaemic heart failure as underlying cause prior to HTx (*p* = 0.004) and more often presented with fever (*p* = 0.047). Although not statistically significantly different, patients with a history of cellular rejection one year or longer prior to COVID-19, tended to more frequently suffer from a more severe COVID-19 (*p* = 0.078) (Tab. 1).

As expected, the dosage of immunosuppressive medication was lowered more frequently in hospitalised patients (moderate or severe COVID-19) compared with non-hospitalised patients with mild COVID-19 (76% vs 18%, *p* < 0.001), to stimulate viral clearance. The immunosuppressive mediation dosage was gradually increased to normal towards the end of hospitalisation or in the outpatient clinic during the first week after discharge, depending on the white blood cell count. Cardiovascular risk factors (Tab. 1), immunosuppressive medication use (Tab. 1), cardiovascular medication (see Table S1 in Electronic Supplementary Material) and laboratory findings at presentation (see Table S1 in Electronic Supplementary Material) were not different between the subgroups (*p* > 0.05).

### In-hospital characteristics

Hospitalisation was required in 21 HTx patients (38.9%), of whom 7 (33%) had an indication for ICU admission. All hospitalised patients required oxygen suppletion, and ICU admission was indicated in case of respiratory insufficiency despite oxygen suppletion. Median length of hospital stay was 8 days (IQR 4–14) and this was significantly different for the group with moderate COVID-19 (median 6 days, IQR 3–10) and the group with severe disease (median 15 days, IQR 8–22) (*p* = 0.038). Critically ill patients requiring ICU admission were admitted to the ICU for a median duration of 8 days (IQR 6–17), after which they could be transferred to the ward. One patient was not accepted for ICU admission due to comorbidities and therefore stayed in the general ward with conservative treatment.

In-hospital characteristics are also shown in Tab. 1. Body mass index (BMI) (*p* = 0.01) and ischaemic heart failure as underlying cause prior to HTx (*p* = 0.017) were more prevalent in patients with severe COVID-19 compared with patients with moderate disease; the same was true for fever at initial presentation (See Table S1 in Electronic Supplementary Material) (*p* = 0.024). Moreover, although not statistically different, patients with severe COVID-19 tended to be older and time from HTx was longer compared with those with moderate disease. Not surprisingly, lowering of immunosuppressive medication dosage occurred more frequently in severely affected patients compared with patients with moderate COVID-19 (86% vs 71%). Antiviral medication was significantly more frequently prescribed in patients with severe disease compared with those in the moderate disease group (*p* = 0.017).

### Outcomes including post-COVID-19 symptoms

During the 15-month study period, 3 patients (6%) died, all due to COVID-19 (Tab. 2). This accounted for an in-hospital mortality of 14% and an ICU-related mortality rate of 43%. None of the ambulant patients died from COVID-19.

**Table 2:** Outcomes stratified by severity of COVID-19

	Total	Severity of COVID-19		
Outcome	Patients (*n* = 54)	Mild (*n* = 33)	Moderate (*n* = 14)	Severe (*n* = 7)
Death	3 (6)	0	0	3 (43)
Deep venous thrombosis	0	0	0	0
Pulmonary embolism	1 (2)	0	1 (7)	0
Transplant rejection	1 (2)	0	0	1 (14)
Myocardial infarction	1 (2)	1 (3)	0	0

Data are *n* (%)

All three deceased patients had severe COVID-19 and died within 10 days after hospital admission. Only one patient was not admitted to the ICU due to severe comorbidities and died in the ward. Another patient who was partially vaccinated died of COVID-19 in the ICU.

Furthermore, during COVID-19 the following complications occurred in three other patients: pulmonary embolism, cellular rejection and myocardial infarction (MI) (Tab. 2). One patient developed a pulmonary embolism after admittance to the ward, despite treatment with low-molecular-weight heparin. After 6 days in the ward, the patient was successfully discharged and was switched from low-molecular-weight heparin to an oral factor Xa inhibitor. The patient who showed signs of transplant rejection suffered from severe COVID-19 for which low levels of immunosuppression were required; this was probably the cause of the observed transplant rejection episode. Finally, the patient who suffered from MI had a known history of CAV. As the MI occurred after COVID-19, this was thought to be a ‘bystander effect’.

With regard to post-COVID-19 symptoms, data were available for 41 cases, of whom 16 (39%) had persistent symptoms of COVID-19, with a median duration of 30 days (IQR 30–83). All post-COVID-19 symptoms are shown in Tab. 3.

**Table 3:** Post-COVID-19 symptoms

		Severity of COVID-19			
Symptom	Patients (*n* = 41)	Mild (*n* = 26)	Moderate (*n* = 12)	Severe (*n* = 3)	*P*-value^a^
Fatigue	8 (20)	5 (19)	3 (25)	0	0.393
Declined exercise tolerance	5 (12)	3 (12)	2 (17)	0	0.574
Anosmia	1 (2)	0	1 (6)	0	–
Agitation	1 (2)	0	0	1 (33)	–

Data are *n* (%)

^a^*P*-value for difference between mild and moderate disease groups

## Discussion

In this retrospective observational study, we examined the characteristics and outcomes of 54 HTx patients with COVID-19 in the Netherlands, where routine testing for SARS-CoV‑2 and contact tracing are available. Compared with the general population, we found that HTx patients were at increased risk of complicated COVID-19, illustrated by a large need for hospitalisation (1.6% vs 38.9%) [1]. Although our ICU admission rate (33%) was in line with those in previous studies on HTx patients, our hospitalisation (39%), in-hospital mortality (14%) and all-cause mortality rates (6%) were substantially lower than most that have been previously described (see Table S2 in Electronic Supplementary Material) [2, 7–15].

A possible explanation for the lower hospitalisation and all-cause mortality rates is the nationwide availability of routine COVID-19 testing in the Netherlands. At least 32 patients (59%) in our study did not present in an emergency department but contacted the cardiology department to notify they had been tested positive for SARS-CoV‑2 by the Dutch Municipal Health Service (*GGD*) per protocol. This suggests that previously reported mortality rates are an overestimation due to selection bias, as those patients were mostly included upon presentation in the emergency department. Another explanation could be that during the COVID-19 pandemic, the Municipal Health Service in the Netherlands provided contact tracing. This led to diagnosing mild COVID-19 cases that may have gone undiagnosed in previous studies. Furthermore, in comparison with other studies, our cohort was younger, had a longer time since transplant, included fewer cases of diabetes mellitus and had a lower BMI [8, 11], which indicated a more favourable risk profile and could further explain the lower mortality rate we observed. Lastly, our population predominantly consisted of COVID-19 during the second wave of the COVID-19 pandemic in the Netherlands (Fig. 1), which might be associated with a lower mortality rate compared with infected transplant recipients during the first wave [13].

Recent studies investigating organ transplant recipients have established that HTx recipients have similar COVID-19 outcomes as the overall transplantation population [2, 11–14]. Additionally, lung transplantation recipients have the highest overall mortality rate compared with other transplant recipients (see Table S3 in Electronic Supplementary Material) [2, 11–14]. Moreover, our data suggested that the prevalence of post-COVID-19 symptoms is comparable with that seen in the general population (61% vs 55–64%) [19, 20].

When comparing non-hospitalised with hospitalised HTx patients with COVID-19, older age and ischaemic heart failure as underlying cause prior to HTx were seen more frequently in case of a more severe COVID-19. Furthermore, higher BMI and ischaemic heart failure as underlying cause prior to HTx were more prevalent in patients with severe COVID-19 compared with patients with moderate COVID-19. Age at time of infection has been a widely described risk factor for a complicated infection, which could indicate that ‘common’ risk factors for a complicated SARS-CoV‑2 infection also apply to the HTx population [21]. It has been proposed that ischaemic heart failure as underlying cause prior to HTx is more prevalent in patients with more severe disease progression because of the vasculopathies and additional comorbidities seen in these patients [22].

Interestingly, we observed a trend towards more severe COVID-19 in patients with a history of transplant rejection. Differences in HLA subset or medication compliance could play a role, as well as variation in immunosuppressive therapeutic levels beyond routine measurements during outpatient consultations. Up till now, the precise immunological processes involved remain a matter of debate [23].

Although vaccination against COVID-19 has been available in the Netherlands since March 2021, hospitalisation was required in 4 out of 5 HTx patients whom had been (partially) vaccinated. This is in line with the growing body of evidence showing that both humoral and cellular immune responses are significantly impaired in solid-organ transplant recipients [4–6].

### Study limitations

The main limitations of our study are its retrospective nature and the lack of a standard approach or formal guidelines for HTx patients, making differences in clinical practice between the Dutch transplant centres inevitable. Nevertheless, these potential differences are at the same time a strength of the current analysis, as it is a real-world study including all HTx recipients with proven COVID-19 in the Netherlands.

Furthermore, although we tried to limit selection bias as much as possible by including patients outside of hospital, asymptomatic HTx patients with COVID-19 could have remained undetected. Since hospitals are required to contact the transplantation centre in case of admission of a transplant recipient, it is unlikely that additional HTx patients with hospitalisation- or COVID-19-related mortality were missed. Importantly, if asymptomatic SARS-CoV-2-positive transplant recipients were included in the current analysis, this would have led to even lower hospitalisation and mortality rates. Of note, the assumption was made that all HTx patients informed their treating physician correctly when they had a positive SARS-CoV‑2 test result.

## Conclusion

This multicentre, retrospective, observational study showed that HTx patients comprise a high-risk population for developing complicated COVID-19. Compared with previous studies, the all-cause mortality rate in our cohort was substantially lower.

## Supplementary Information

**Table S1** Diagnostics, vaccination status, symptoms, laboratory results and cardiovascular medication at presentation

**Table S2** Hospitalisation and mortality rates in previous studies

**Table S3** Hospitalisation and mortality rates in previous studies, stratified by type of transplantation
