# Supplementary material for: Characteristics and outcomes of COVID-19 in heart transplantation recipients in the Netherlands
Source: Neth Heart J. 2022 Sep 8;30(11):519–25. doi: 10.1007/s12471-022-01720-9 (PMC9454385; doi:10.1007/s12471-022-01720-9)
Supplement: Supplementary file 1 — Table S1 Diagnostics, vaccination status, symptoms, laboratory results and cardiovascular medication at presentation [file 12471_2022_1720_MOESM1_ESM.docx]

**Table S1** Diagnostics, vaccination status, symptoms, laboratory results and cardiovascular medication at presentation

|  | **Total** | **Severity of COVID-19** | | |
| --- | --- | --- | --- | --- |
| **Variable** | **Patients (*n* = 54)** | **Mild (*n* = 33)** | **Moderate (*n* = 14)** | **Severe (*n* = 7)** |
| COVID-19 proven by rt-PCR (N (%)) | 52 (96) | 31 (94) | 14 (100) | 7 (100) |
| COVID-19 proven by serology (N (%)) | 2 (4) | 2 (6) | 0 (0) | 0 (0) |
| *Vaccinated* |  |  |  |  |
| Yes (N (%)) | 3 (6) | 0 (0) | 2 (14) | 1 (14) |
| Partially (N (%)) | 2 (4) | 1 (3) | 0 (0) | 1 (14) |
| No (N (%)) | 45 (83) | 30 (91) | 11 (79) | 4 (57) |
| Unknown (N (%)) | 4 (7) | 2 (6) | 1 (7) | 1 (14) |
| *Symptoms at initial presentation* |  |  |  |  |
| Upper airway complaints (yes)  (N (%)) | 34 (63) | 19 (58) | 11 (79) | 4 (57) |
| Fever (yes) (N (%))**^†*^** | 26 (48) | 16 (49) | 4 (29) | 6 (86) |
| Fatigue (yes) (N (%)) | 33 (61) | 16 (49) | 11 (79) | 6 (86) |
| Muscle ache (yes) (N (%)) | 21 (39) | 13 (39) | 5 (36) | 3 (43) |
| Declined exercise tolerance (yes) (N (%)) | 28 (52) | 14 (42) | 9 (64) | 5 (71) |
| Anosmia (yes) (N (%)) | 8 (15) | 7 (21) | 1 (7) | 0 (0) |
| GI-symptoms (yes) (N (%)) | 12 (22) | 5 (15) | 4 (29) | 3 (43) |
| *Laboratory at presentation* |  |  |  |  |
| Haemoglobin (mmol/L) (SD) (n=22) | 8.2 (1.0) | 8.3 (0.6) | 8.5 (1.0) | 7.5 (1.2) |
| Thrombocytes (*10^9/L) (SD) (n=22) | 205 (78) | 215 (89) | 207 (85) | 187 (58) |
| Leukocytes (*10^9/L) (SD) (n=22) | 6.2 (2.3) | 5.6 (2.1) | 6.5 (2.5) | 6.4 (2.7) |
| Urea (mmol/L) (SD) (n=17) | 17 (32) | 6.0 (1.6) | 12 (9) | 42 (65) |
| Creatinine (umol/L) (SD) (n=22) | 128 (69) | 107 (42) | 150 (81) | 102 (69) |
| ASAT (U/L) (SD) (n=19) | 30 (13) | 32 (19) | 28 (10) | 33 (7) |
| Bilirubin (umol/L) (SD) (n=10) | 11 (6.8) | 7 | 12.6 (9.4) | 9.3 (2.2) |
| NT-proBNP (pg/mL) (SD) (n=6) | 322 (218) | 65 (66) | 430 (123) | 510 |
| *Cardiovascular medication* |  |  |  |  |
| Beta-blockers (N (%)) | 19 (35) | 12 (36) | 4 (29) | 3 (43) |
| ACE-i/ARB (N (%)) | 32 (59) | 20 (61) | 8 (57) | 4 (57) |
| ARNI (N (%)) | 1 (2) | 0 (0) | 1 (7) | 0 (0) |
| Ca-antagonist (N (%)) | 28 (52) | 17 (52) | 8 (57) | 3 (43) |
| Aldosterone receptor antagonist (N (%)) | 6 (11) | 2 (6) | 2 (14) | 2 (29) |
| Statin (N (%)) | 44 (82) | 28 (85) | 11 (79) | 5 (71) |

*ACE-i* angiotensin converting enzyme inhibitor, *ARB* angiotensin receptor blocker, *ARNI* angiotensin receptor neprilysin inhibitor, *ASAT* Aspartate transaminase, *COVID-19* coronavirus disease 2019, *GI* gastro-intestinal, *rt-PCR* reverse-transcription polymerase chain reaction, *NT-proBNP* N-terminal prohormone of brain natriuretic peptide, *SD* standard deviation

**^†^** used for comparison between mild, moderate and severe disease

^*^ used for comparison between moderate and severe disease

**^†^** 0.05 > p ≥ 0.01

^*^0.05 > p ≥ 0.01
