# Supplementary material for: Characteristics and outcomes of COVID-19 in heart transplantation recipients in the Netherlands
Source: Neth Heart J. 2022 Sep 8;30(11):519–25. doi: 10.1007/s12471-022-01720-9 (PMC9454385; doi:10.1007/s12471-022-01720-9)
Supplement: Supplementary file 2 — Table S2 Hospitalisation and mortality rates in previous studies [file 12471_2022_1720_MOESM2_ESM.docx]

**Tab. S2** Hospitalisation and mortality rates in previous studies

|  | Number of patients | Hospitalization rate | ICU admission | In-hospital mortality | All-cause mortality |
| --- | --- | --- | --- | --- | --- |
| Coll et al.[2] | 59 | 51 (86%) | 7 (14%) | Not reported | 13 (22%) |
| Latif et al.[7] | 28 | 22 (79%) | Not reported | 7 (32%) | 7 (25%) |
| Genuardi et al.[8] | 99 | 63 (68%) | 24 (38%) | 15 (24%) | 15 (16%) |
| Bottio et al. [9] | 53 | 39 (74%) | 4 (10%) | 14 (36%) | 14 (26%) |
| Marcondes-Braga et al.[10] | 40 | 33 (83%) | 17 (52%) | 11 (65%) | 11 (28%) |
| Hadi et al.[11] | 183 | 66 (36%) | 26 (39%) | Not reported | 13 (7%) |
| Felldin et al.[12] | 5 | 3 (60%) | Not reported | 1 (33%) | 1 (20%) |
| Coll et al.[13]* | 944 | 41 (49%) | 8 (20%) | Not reported | 12 (14%) |
| Kates et al.[14] | 57 | 47 (83%) | 18 (38%) | Not reported | 8 (14%) |
| Rivinius et al.[15] | 21 | 19 (91%) | Not reported | 7 (37%) | 7 (33%) |

*ICU* intensive care unit

* Only second wave included. First wave already included in Coll et al.[2]
