# Supplementary material for: Characteristics and outcomes of COVID-19 in heart transplantation recipients in the Netherlands
Source: Neth Heart J. 2022 Sep 8;30(11):519–25. doi: 10.1007/s12471-022-01720-9 (PMC9454385; doi:10.1007/s12471-022-01720-9)
Supplement: Supplementary file 3 — Table S3 Hospitalisation and mortality rates in previous studies, stratified by type of transplantation [file 12471_2022_1720_MOESM3_ESM.docx]

**Table S3** Hospitalisation and mortality rates in previous studies, stratified by type of transplantation

|  | Coll et al.[2] | Hadi et al.[11] | Felldin et al.[12] | Coll et al.[13]* | Kates et al.[14] |
| --- | --- | --- | --- | --- | --- |
| **Hospitalization rate** |  |  |  |  |  |
| Overall | 581 (89%) | 715 (31%) | 38 (75%) | 501 (51%) | 376 (78%) |
| *Type of transplant* |  |  |  |  |  |
| Heart | 51 (86%) | 66 (36%) | 3 (60%) | 41 (49%) | 47 (83%) |
| Kidney | 338 (90%) | 412 (27%) | 21 (68%) | 344 (55%) | 254 (80%) |
| Liver | 67 (88%) | 71 (30%) | 6 (75%) | 70 (44%) | 47 (64%) |
| Lung | 48 (96%) | 71 (30%) | 5 (100%) | 40 (77%) | 24 (80%) |
| Pancreas | 4 (67%) | - | - | 6 (32%) | - |
| Multivisceral | 0 (0%) | 97 (42%) | 3 (75%) | - | - |
| Allogenic hematopoietic stem cell transplant | 45 (80%) | - | - | - | - |
| Autologous hematopoietic stem cell transplant | 28 (97%) | - | - | - | - |
| **ICU admission** |  |  |  |  |  |
| Overall | 84 (14%) | 253 (35%) | Not reported | 115 (23%) | 163 (43%) |
| *Type of transplant* |  |  |  |  |  |
| Heart | 7 (14%) | 26 (39%) | Not reported | 8 (10%) | 18 (38%) |
| Kidney | 57 (17%) | 137 (33%) | Not reported | 81 (13%) | 107 (42%) |
| Liver | 3 (4%) | 25 (35%) | Not reported | 12 (8%) | 23 (49%) |
| Lung | 6 (13%) | 20 (28%) | Not reported | 14 (27%) | 12 (50%) |
| Pancreas | 2 (50%) | - | - | 0 (0%) | - |
| Multivisceral | 0 (0%) | 39 (40%) | Not reported |  | - |
| Allogenic hematopoietic stem cell transplant | 4 (9%) | - | - | - | - |
| Autologous hematopoietic stem cell transplant | 5 (19%) | - | - | - | - |
| **All-cause mortality** |  |  |  |  |  |
| Overall | 174 (27%) | 110 (5%) | 5 (9%) | 144 (17%) | 90 (19%) |
| *Type of transplant* |  |  |  |  |  |
| Heart | 13 (22%) | 13 (7%) | 1 (20%) | 12 (14%) | 8 (14%) |
| Kidney | 103 (28%) | 57 (4%) | 3 (10%) | 90 (14%) | 57 (18%) |
| Liver | 17 (22%) | Not reported | 1 (13%) | 30 (19%) | 15 (17%) |
| Lung | 23 (46%) | Not reported | 0 (0%) | 22 (42%) | 10 (33%) |
| Pancreas | 0 (0%) | - | - | 1 (5%) | - |
| Multivisceral | 0 (0%) | 16 (7%) | 0 (0%) | 0 (0%) | - |
| Allogenic hematopoietic stem cell transplant | 11 (20%) | - | - | - | - |
| Autologous hematopoietic stem cell transplant | 7 (24%) | - | - | - | - |

*ICU* intensive care unit

**^*^** Only second wave included. First wave already included in Coll et al.[2]
